# Supplementary material for: A spontaneous mitonuclear epistasis converging on Rieske Fe-S protein exacerbates complex III deficiency in mice
Source: Nat Commun. 2020 Jan 16;11:322. doi: 10.1038/s41467-019-14201-2 (PMC6965120; doi:10.1038/s41467-019-14201-2)
Supplement: Supplementary file 1 — Supplementary Information [file 41467_2019_14201_MOESM1_ESM.pdf]

Supplementary information

A spontaneous mitonuclear epistasis converging on Rieske Fe-S protein exacerbates complex III deficiency in mice

Purhonen et al.

Supplementary Table 1. A mtDNA variant and seven homozygous nDNA coding-region variants identified from C57BL/6JBomTac-derived in-house strain using whole genome sequences (C57BL/6JCrl as reference)

Gene	Full name	Identifier	Type of variant	Chromosome	Exon	Nucleotide	Amino acid
mt-Cyb	Cytochrome b	ENSMUSG00000064370	Nonsynonymous	mtDNA		G760A	D254N
Prex2	Phosphatidylinositol-3,4,5-Trisphosphate-Dependent Rac Exchange Factor 2	ENSMUSG00000048960	Nonsynonymous	1	6	G533A	R178Q
Taar2	Trace Amine Associated Receptor 2	ENSMUSG00000059763	Nonsynonymous	10	2	C685A	R229S
Asrgl1	Asparaginase Like 1	ENSMUSG00000024654	Stop-gain	19	5	597G	Y199X
Kras	Kirsten rat sarcoma viral oncogene homolog	ENSMUSG00000030265	Synonymous	6	3	T96C	Y32Y
Rhox6	Reproductive homeobox 6	ENSMUSG00000006200	Synonymous	X	2	C153T	L51L
B230364G03Rik	Antisense overlapping with neuromedin B receptor	ENSMUSG00000096963	Frameshift insertion	10	1	8_9insTTTA	L3fs
B430203G13Rik-201		ENSMUSG00000067356	Frameshift insertion	12	1	64_65insCTTT	L22fs

Supplementary Table 2. Properties of tested *R. capsulatus* strains

Bacterial strain	Growth under photosynthetic conditions [a]	Enzymatic activity [b] **[s^{-1}]**	Reactive oxygen species production by the antimycin -inhibited enzyme
WT	Ps^+	140 ± 5	17%
D278N	Ps^+	140 ± 6	18%

[a] Ps+ indicates photosynthetic competence

[b] Mean and S.D. of at least five measurements

WT reference values are from Borek et al. 2015[67].

a

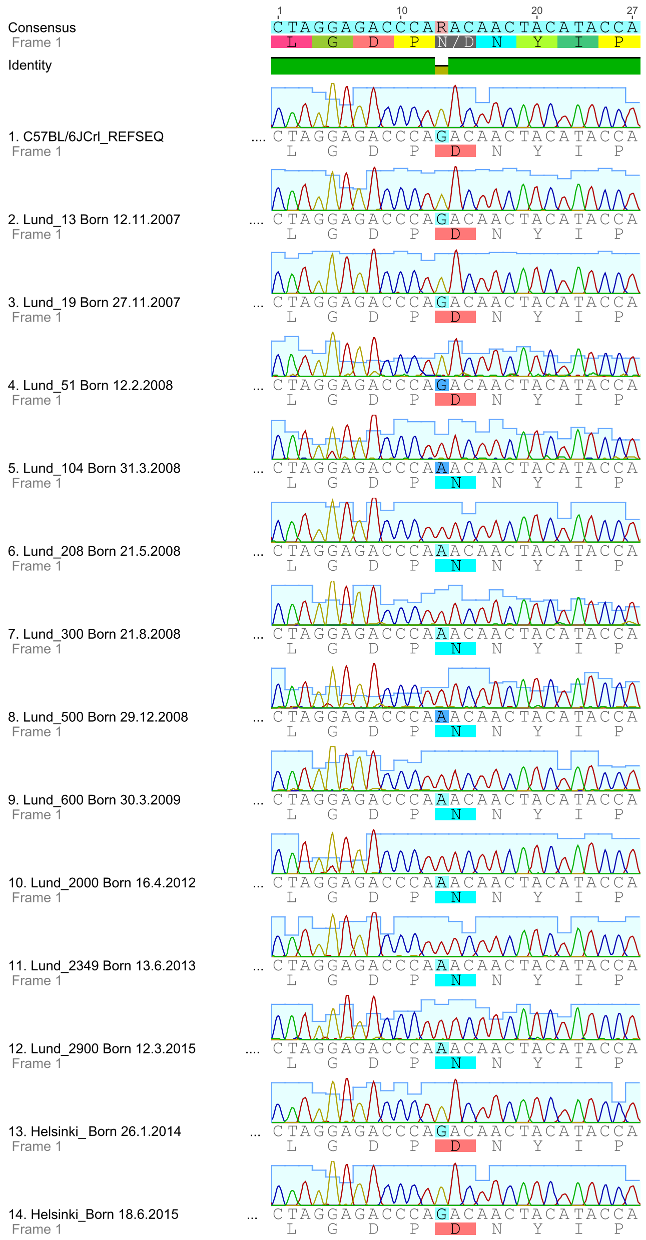

b

Maternal background	Paternal background	Mouse ID	Tissue	No. of clones	WT	D254N
C57BL/6JBomTac*	C57BL/6JBomTac*	LU2921	kidney	50	0	50
		LU2921	skeletal muscle	50	0	50
		LU2921	heart	50	0	50
C57BL/6JBomTac*	C57BL/6JCrl	LU164	liver	50	0	50
		LU164	kidney	50	0	50
		LU164	skeletal muscle	46	0	46
		LU164	heart	50	0	50
C57BL/6JCrl	C57BL/6JBomTac*	JKa193	skin	10	10	0

Supplementary Figure 1. Appearance of the *m.G14904A* (*mt-Cyb*$^{c.G760A}$, *mt-Cyb*$^{p.D254N}$) haplotype in the in-house Lund C57BL/BomTac colony, and analysis of its heteroplasmy. a, Representative Sanger sequencing chromatograms from DNA collected between 2007 and 2015. Altogether 76 mice from the colony were genotyped for the *mt-Cyb* variant from archived genotyping samples. **b**, Evaluation of heteroplasmy in somatic tissues of one mouse from the Lund colony and one F1 hybrid mouse with a Lund colony-derived C57BL/6JBomTac maternal parent. *mt-Cyb* PCR amplicons from several tissues were cloned in bacteria and the resulting plasmids sequenced.

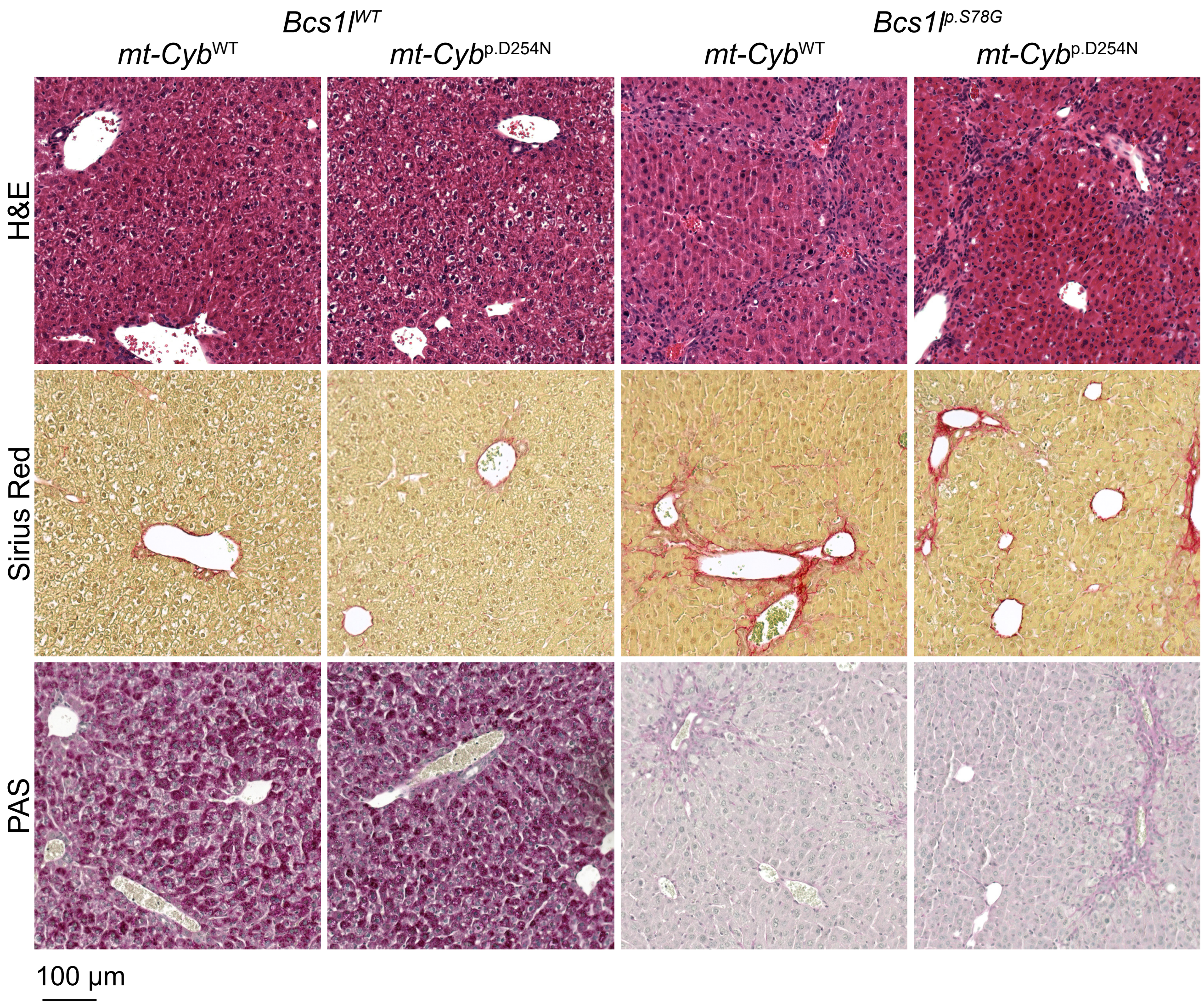

Supplementary Figure 2. CIII deficiency-related hepatic histopathology in the two mtDNA backgrounds prior to end-stage disease. a, H&E, collagen (Sirius Red) and glycogen (PAS) stains showing hepatopathy with incipient fibrosis and glycogen depletion in the *Bcs1l*$^{p.S78G}$ mice between P29 and P33 with and without *mt-Cyb*$^{p.D254N}$.

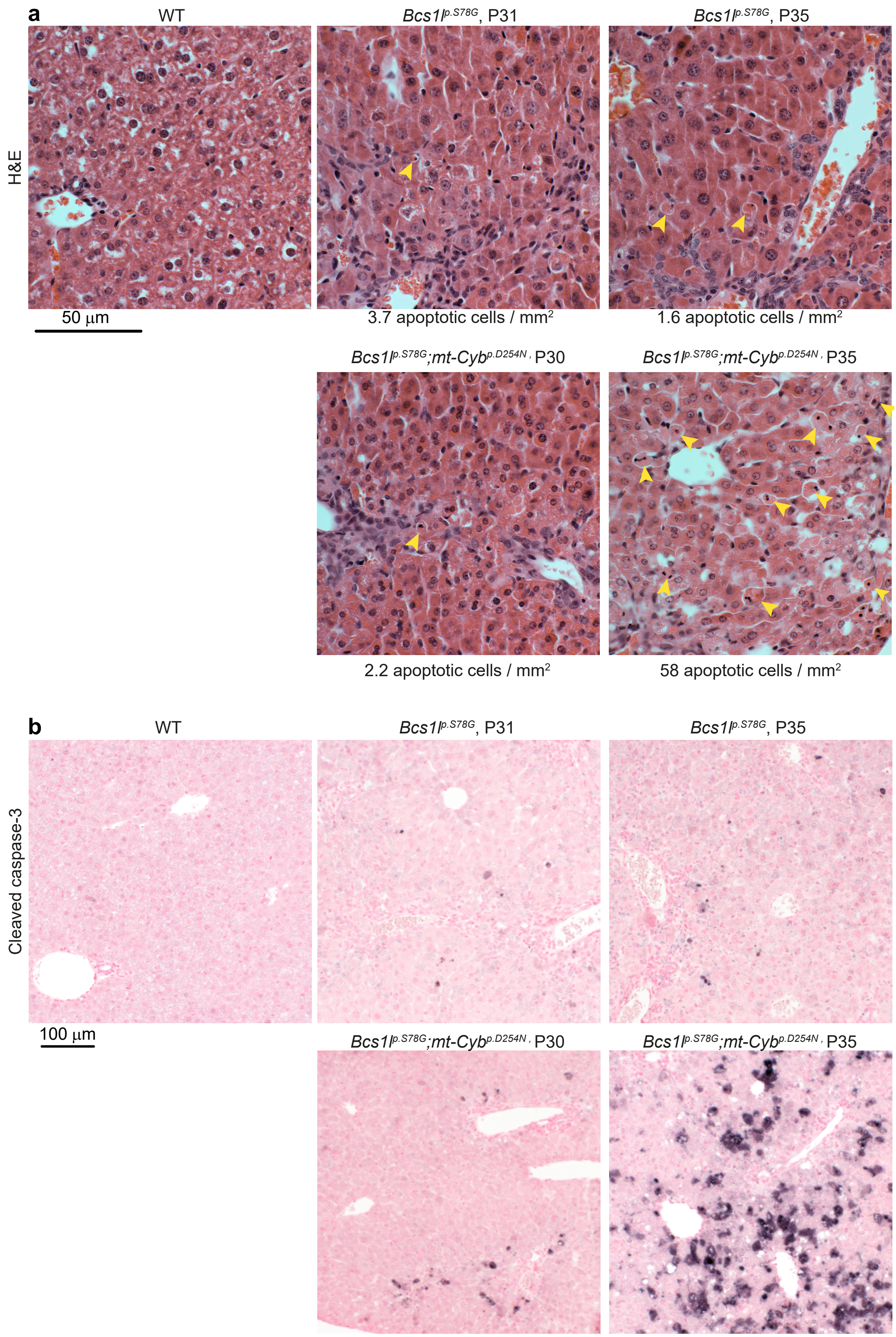

Supplementary Figure 3. Widespread hepatocyte death in *Bcs1l$^{p.S78G}$* mice carrying *mt-Cyb$^{p.D254N}$*. a, H&E-stained liver sections with arrow heads pointing to apoptotic bodies: eosinophilic cytoplasm surrounded by halo of empty space, and fragmented or condensed nucleus. **b,** Visualization of apoptotic cells by immunostaining for cleaved caspase 3.

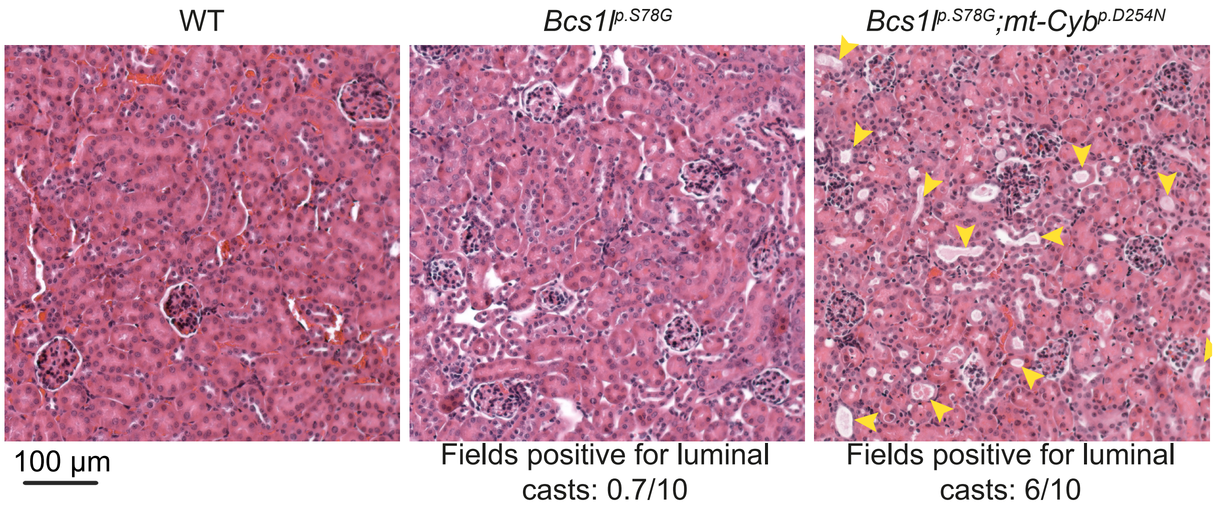

Supplementary Figure 4. Effect of *mt-Cyb*$^{p.D254N}$ on renal tubulopathy in *Bcs1l*$^{p.S78G}$ mice. H&E-stained kidney sections show increased proximity of glomeruli in *Bcs1l*$^{p.S78G}$ mice, suggesting loss of tubular mass. The kidneys of *Bcs1l*$^{p.S78G}$ mice carrying *mt-Cyb*$^{p.D254N}$ show frequent apoptotic cells and proteinuria-associated tubular casts (arrow heads), both of which were only sporadic in the absence of *mt-Cyb*$^{p.D254N}$.

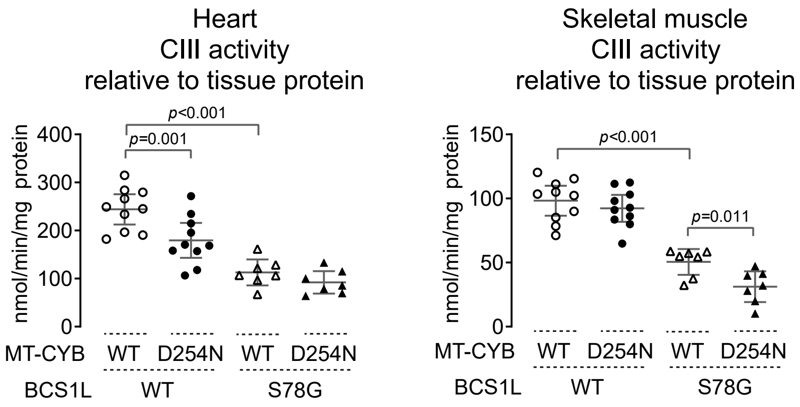

Supplementary Figure 5, CIII activity in cardiac and skeletal muscle (quadriceps) homogenates plotted relative to tissue protein amount. The data are from P30 cohort of mice backcrossed to C57BL/6JCrl. Activity units were calculated by using 18.5 $mM^{-1}cm^{-1}$ extinction coefficient for 550 nm absorbance change and are expressed relative to 600g-supernatant tissue protein (Bradford assay, albumin equivalents).

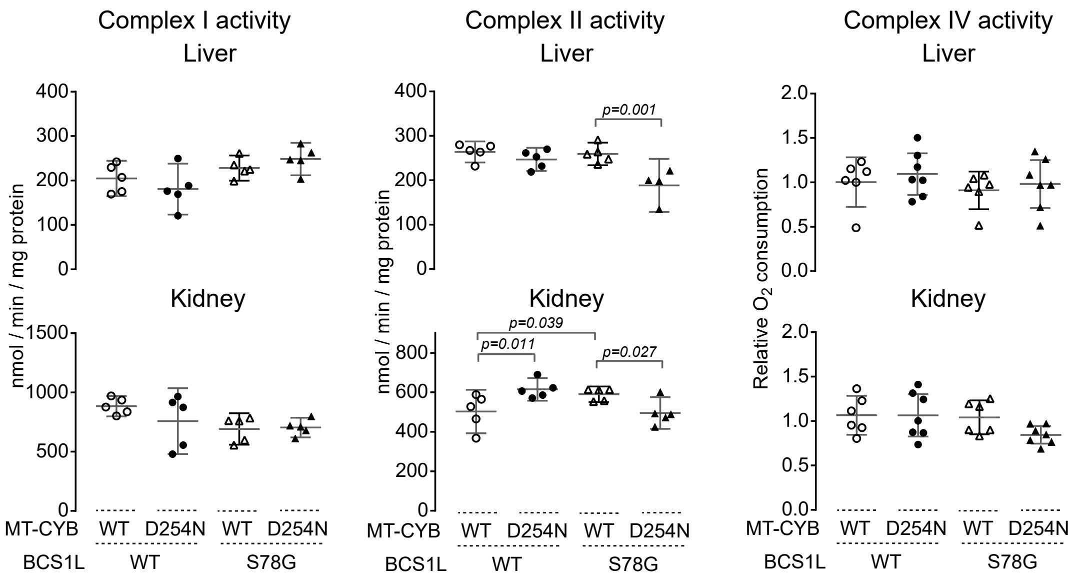

Supplementary Figure 6. Activities of complex I, II and IV in isolated liver and kidney mitochondria from F1 hybrid mice. CI, CII and CIII activities were measured spectrophotometrically and CIV using an oxygraph. Error bars represent 95% CI of mean.

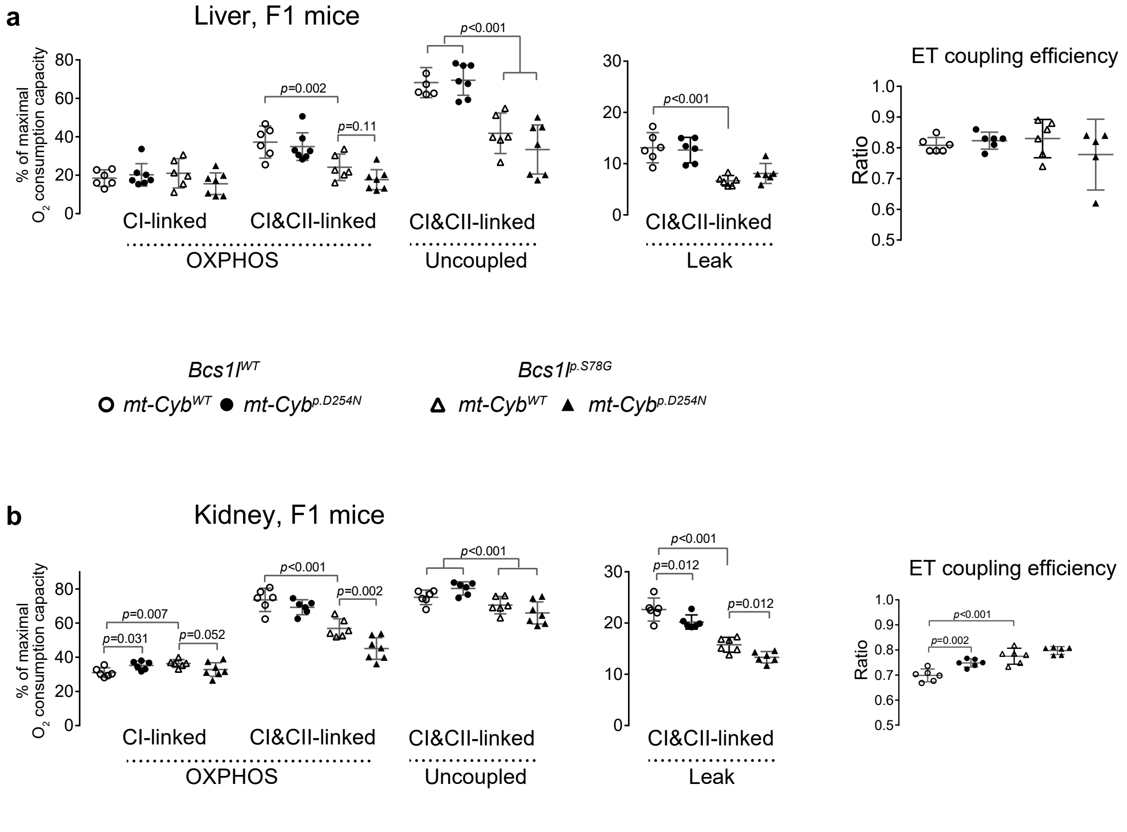

Supplementary Figure 7. Respirometric analysis of liver (a) and kidney (b) mitochondria from F1 hybrid mice. Comparable analyses from a cohort of mice backcrossed to C57BL/6JCrl are shown in Fig. 4 and Supplementary Fig. 8 and 9. The oligomycin concentration and batch used here was later found to somewhat disrupt membrane potential and inhibit maximal electron transfer in liver mitochondria (see main text). Maximal oxygen consumption capacity of cytochrome c oxidase was set as a reference state for the oxygen fluxes. Error bars present 95% CI of mean.

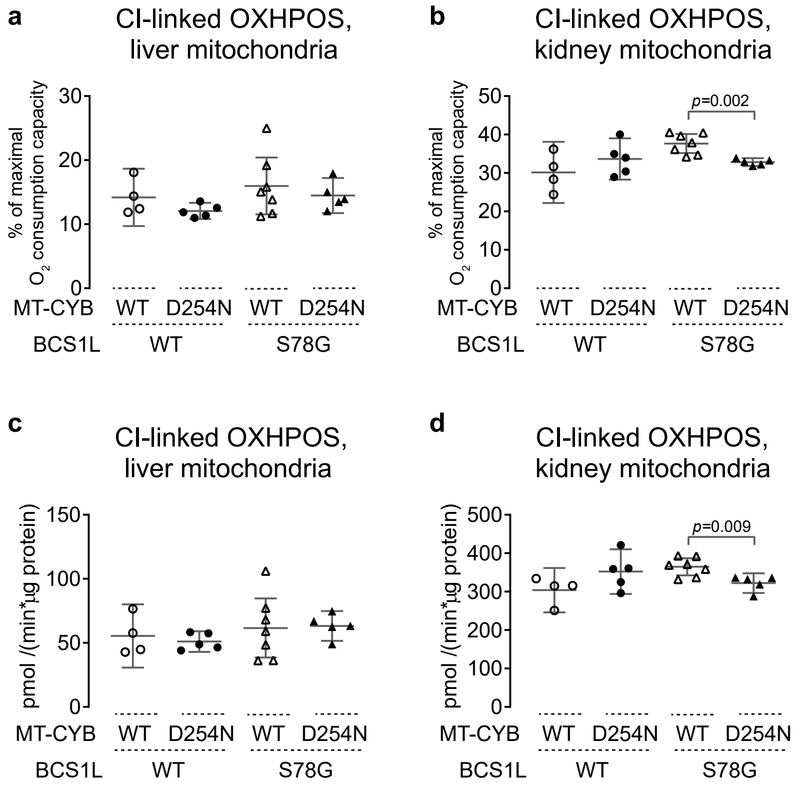

Supplementary Figure 8. CI-linked phosphorylating respiration in mitochondria from mice backcrossed to C57BL/6JCrl. Respiration was measured in the presence of ADP, malate, pyruvate and glutamate to drive mitochondrial NADH oxidation-dependent ATP production. **a-b**, Phosphorylating respiration relative to maximal oxygen consumption capacity (maximal oxygen consumption by CIV). **c-d**, Phosphorylating respiration relative to mitochondrial protein. Error bars present 95% CI of mean.

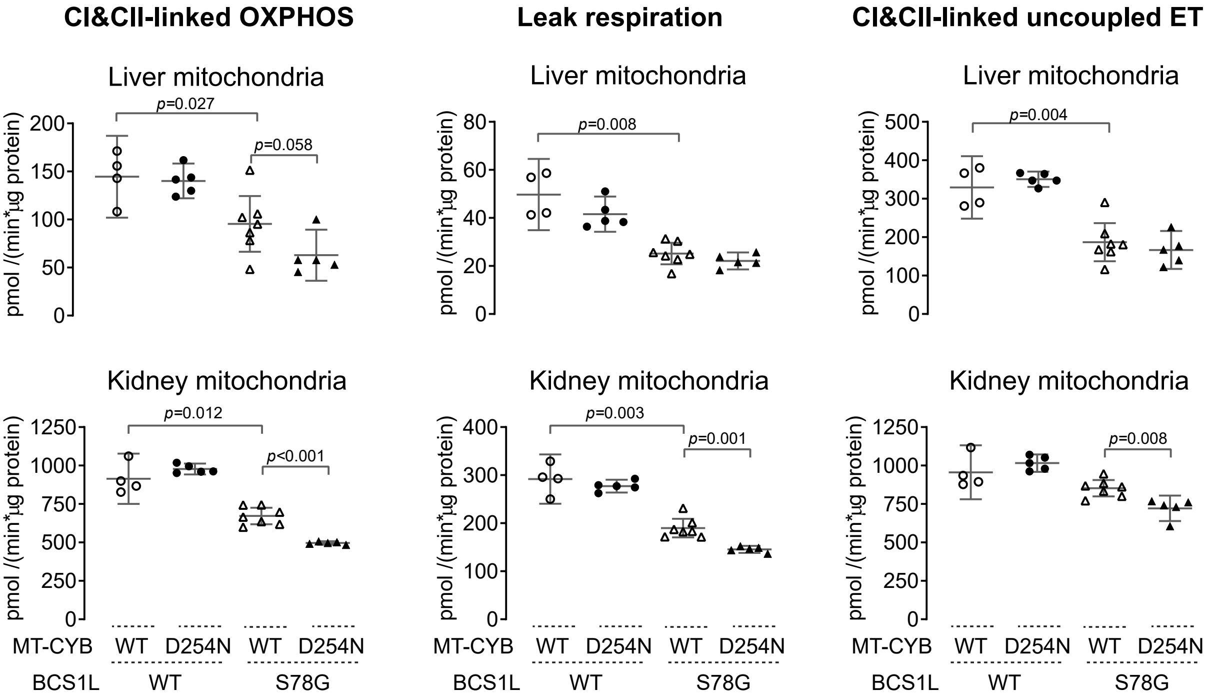

Supplementary Figure 9. Respirometry data (Fig. 4 a-c) plotted relative to mitochondrial protein amount. Error bars represent 95% CI of the mean.

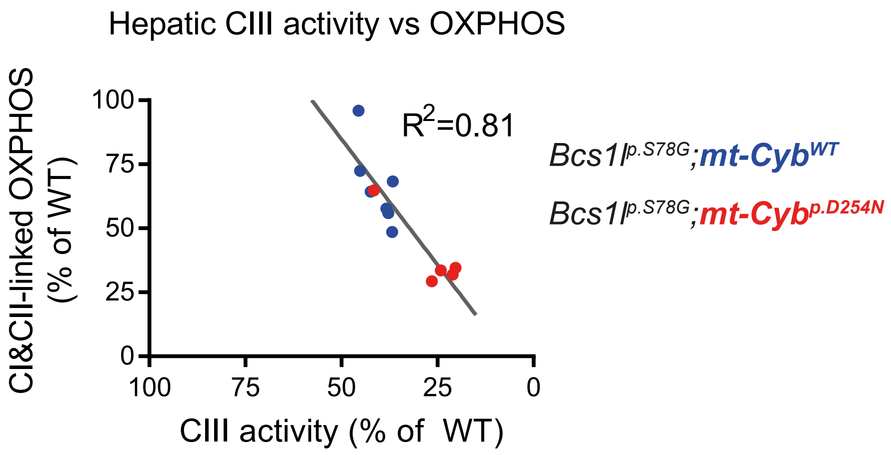

Supplementary Figure 10. Correlation between hepatic CIII activity and maximal OXHPOS capacity. The OXPHOS capacity (Fig. 4a) was assessed by measuring oxygen consumption by freshly isolated mitochondria in presence of malate, pyruvate, glutamate, succinate and ADP. CIII activity, from freeze-thawed mitochondria, was measured spectroscopically by monitoring decylubiquinol-mediated antimycin-sensitive reduction of cytochrome c.

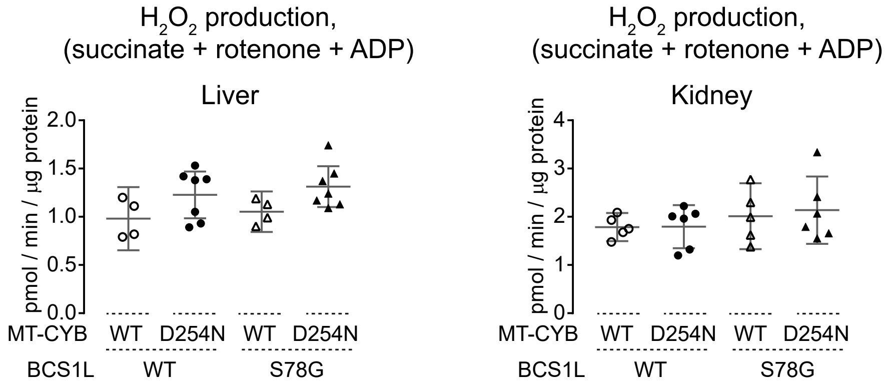

Supplementary Figure 11. H_2O_2 emission by isolated mitochondria respiring with succinate and reverse electron flow blocked by rotenone. Isolated mitochondria were incubated in the presence of the indicated substrates and rotenone, and H_2O_2 production was assessed with Amplex Red-peroxidase assay. The data are from the F1 cohort.

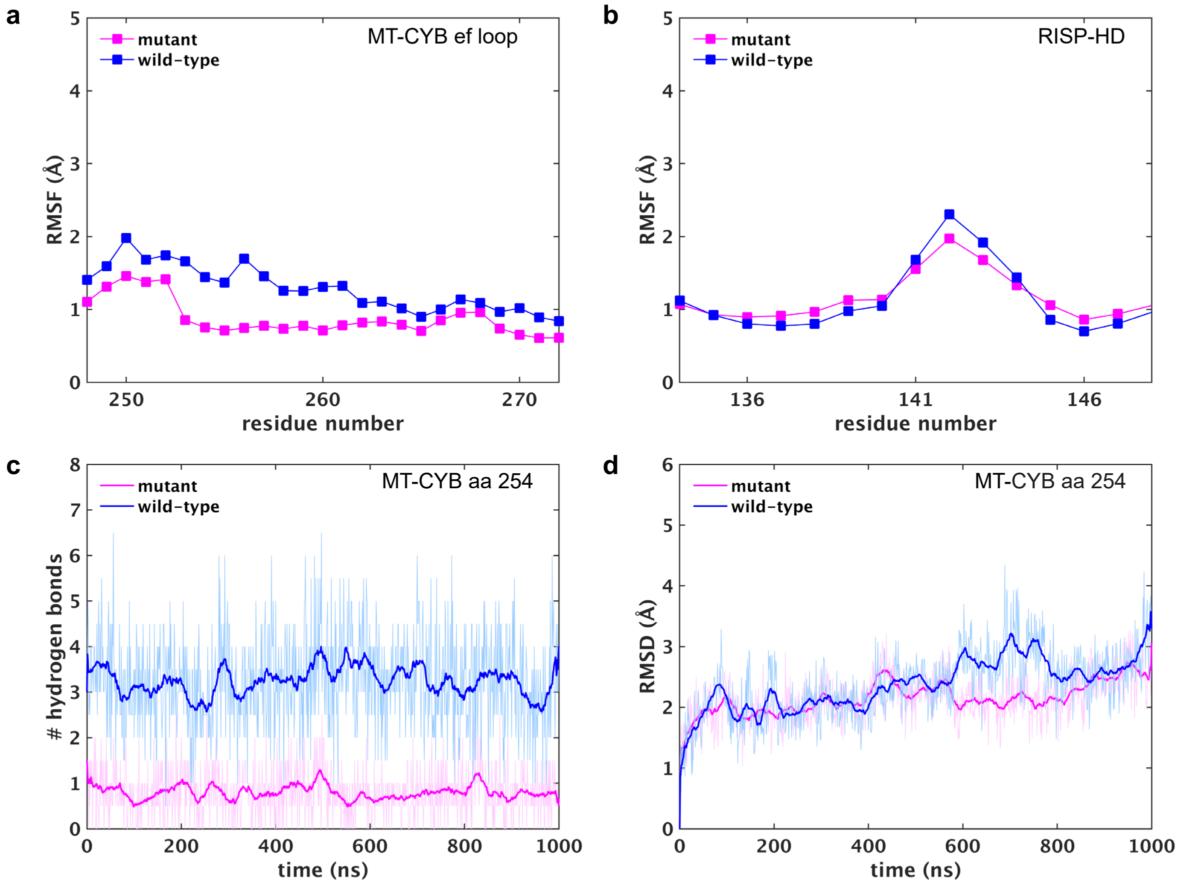

Supplementary Figure 12. Protein dynamics when the Q_o and Q_i sites are occupied by QH_2 and oxidized Q, respectively. a, RMSF of the Cα atoms from segment T250-V270 of the MT-CYB subunit. **b**, RMSF of segment P136-W146 in the RISP subunit. **c**, Number of hydrogen bonds between the sidechain of D254/N254 (D255 in *S. cerevisiae* complex) and water molecules, with a criteria of a 3.0 Å donor-acceptor (D...A) distance and >160º bond angle (D-H....A). **d**, RMSD of the D254/N254 sidechain.

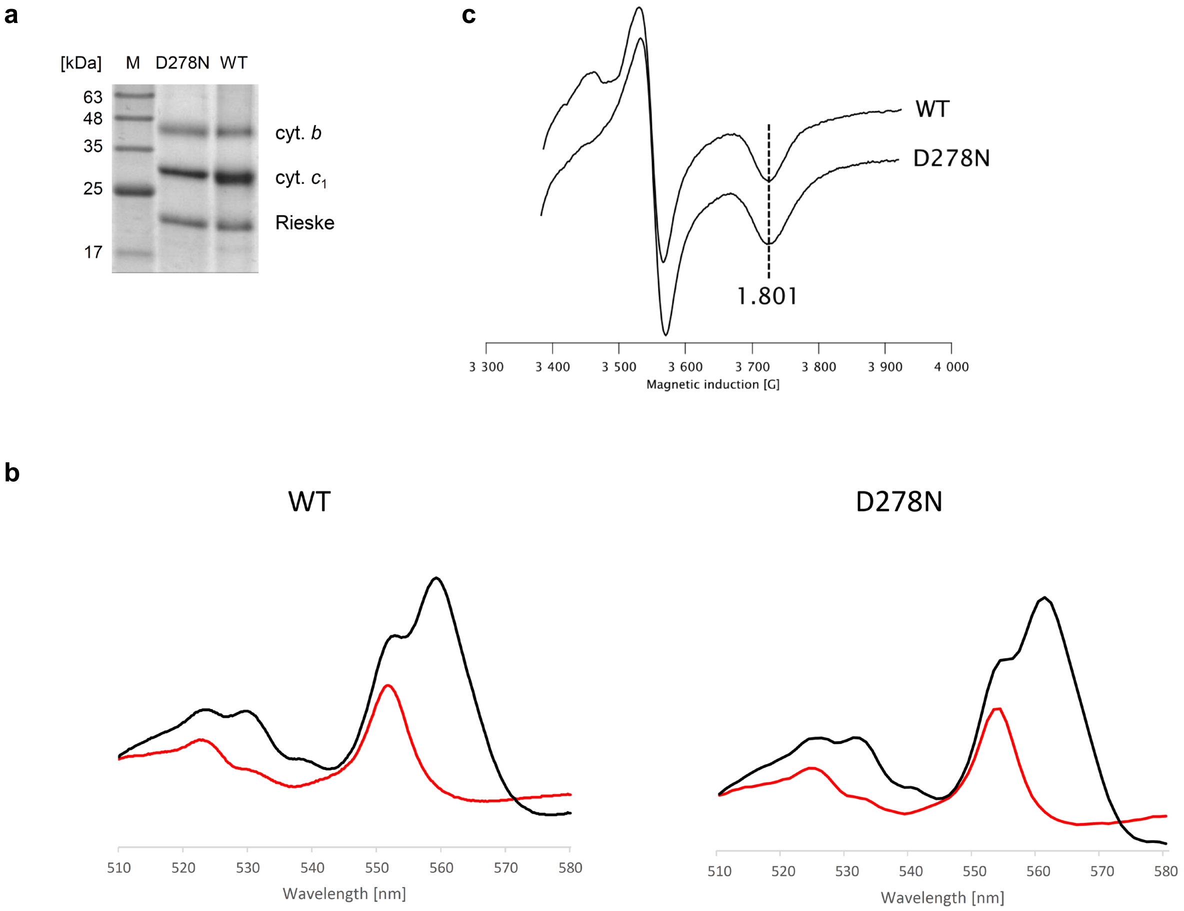

Supplementary Figure 13. Subunit composition and spectral properties of the mutated *Rhodobacter* cytochrome bc_1 complex. **a**, SDS-PAGE analysis of the affinity-purified cytochrome *bc_1* complex containing D278N mutant (corresponding to *M. musculus* D254N) or wild type CYTB. **b**, Optical difference spectra of hemes *b* and *c* of purified wild type cytochrome *bc_1* complex (WT) and D278N) mutant. Red lines represent ascorbate-reduced minus ferricyanide-oxidized cytochrome bc1 spectra (heme c), black lines represent dithionite-reduced minus ferricyanide-oxidized spectra cytochrome bc1 spectra (hemes b and c). **c**, Comparison of X-band continuous wave EPR spectra of [2Fe–2S] cluster of *bc_1* complex in chromatophores isolated from wild type (WT) or D278N strain of *R. capsulatus*.

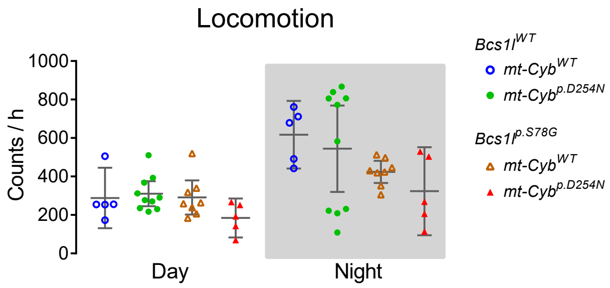

Supplementary Figure 14. Average day and night-time movement counts recorded during indirect calorimetry (Fig.6). Indirect calorimetry cages equipped with infrared beam detectors were used to estimate the physical activity of the mice.

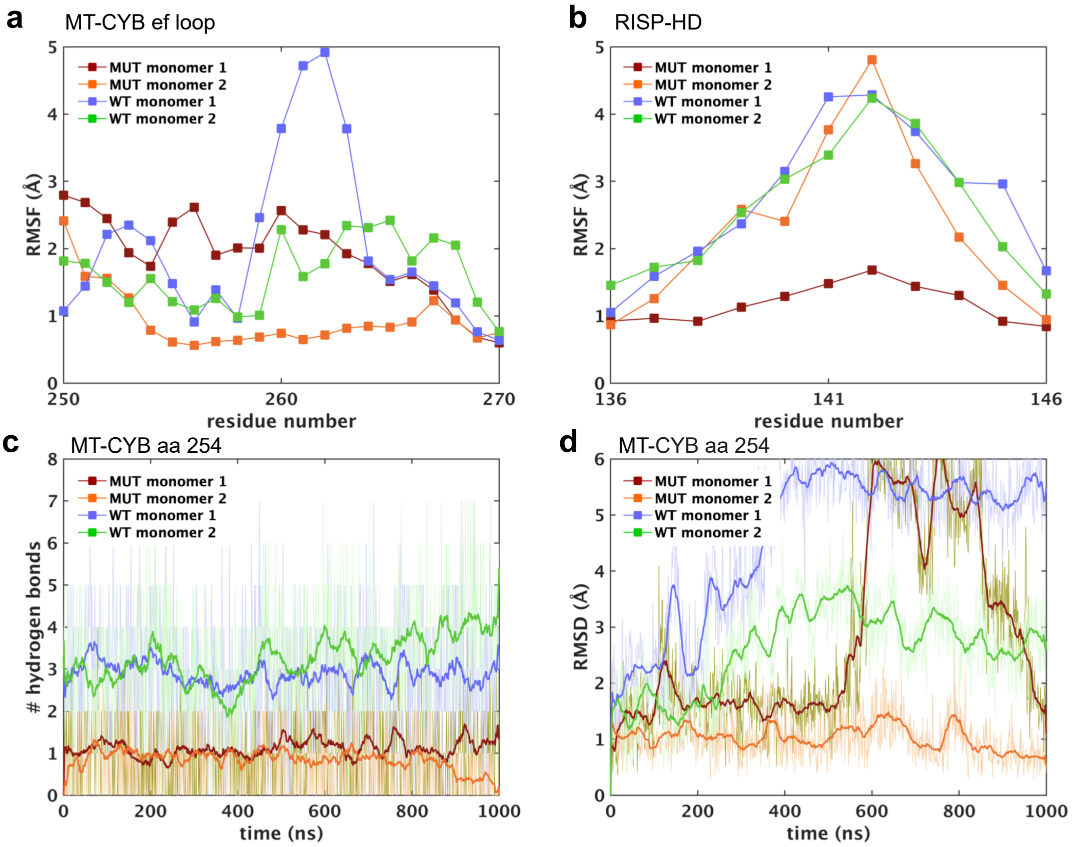

Supplementary Figure 15. Dynamics of segments surrounding D254 from wild-type and mutant without Q molecules modeled. The data from each monomer (1 and 2) is displayed separately, and x- and y-axis labels are same as in Fig. 5. **a**, RMSF of the Cα atoms from segment T250-V270 of the MT-CYB subunit. **b**, RMSF of segment P136-W146 in the RISP subunit. **c**, Number of hydrogen bonds between the sidechain of D254/N254 (D255 in S. cerevisiae complex) and water molecules, with a criteria of a 3.0 Å donor-acceptor (D...A) distance and >160º bond angle (D-H....A). **d**, RMSD of the D254/N254 sidechain. Monomer 1 in the mutant simulation (red-brown trace in panel **a**) show RMSF equivalent to the wild-type case, which is primarily due to the short timescale fluctuation in sidechain of N254 (red-brown trace; 600 – 900 ns in panel **d**) after which it returns to its original conformation. When this part of the simulation trajectory is excluded (only first 500 ns data considered), the RMSF of ef loop segment drops to ca. 1 Å. Overall, despite some differences observed in between the two monomers of a homodimer, in part due to the limited simulation sampling, the data suggest somewhat higher mobility of anionic D254 and segments around it in contrast to neutral mutant N254 (see also Fig. 5 in main text), which may also affect the quinol/quinone binding dynamics.

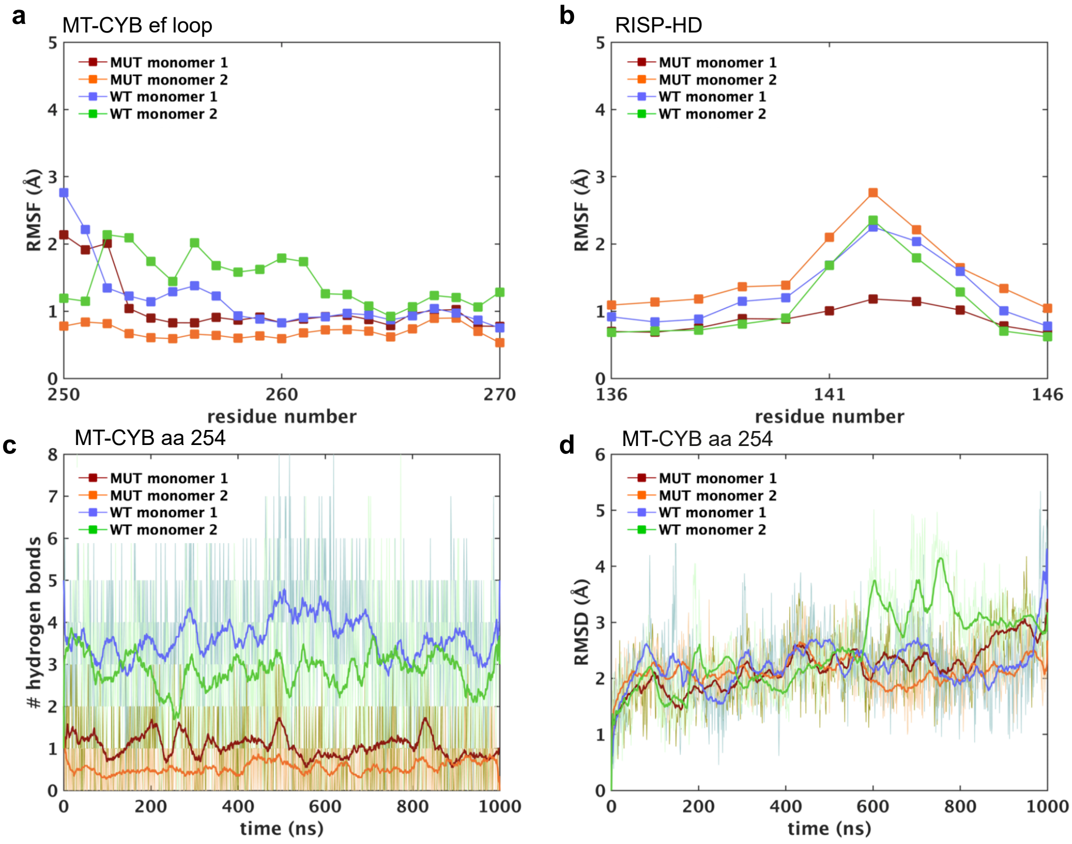

Supplementary Figure 16. Dynamics of segments surrounding D254 from WT and mutant simulations with Q molecules modeled at the Q_o and Q_i sites. The data from each monomer (1 and 2) is displayed separately, and x- and y-axis labels are same as in Fig. 5. In contrast to higher mobility of protein segments in simulations with empty Q_o/Q_i sites, these data reveal overall reduced mobility (see main text). Nevertheless, subtle differences exist between the WT and mutant cases (panels **a** to **d**).
