# Supplementary material for: A spontaneous mitonuclear epistasis converging on Rieske Fe-S protein exacerbates complex III deficiency in mice
Source: Nat Commun. 2020 Jan 16;11:322. doi: 10.1038/s41467-019-14201-2 (PMC6965120; doi:10.1038/s41467-019-14201-2)
Supplement: Supplementary file 3 — Reporting Summary [file 41467_2019_14201_MOESM3_ESM.pdf]

nature research

Corresponding author(s): *Double-blind peer review submissions: write DBPR and your manuscript number here instead of author names.*

Life Sciences Reporting Summary

Nature Research wishes to improve the reproducibility of the work that we publish. This form is intended for publication with all accepted life science papers and provides structure for consistency and transparency in reporting. Every life science submission will use this form; some list items might not apply to an individual manuscript, but all fields must be completed for clarity.

For further information on the points included in this form, see Reporting Life Sciences Research. For further information on Nature Research policies, including our data availability policy, see Authors & Referees and the Editorial Policy Checklist.

Please do not complete any field with "not applicable" or n/a. Refer to the help text for what text to use if an item is not relevant to your study. For final submission: please carefully check your responses for accuracy; you will not be able to make changes later.

▸ Experimental design

1. Sample size

Describe how sample size was determined.

A priori power analysis (80% power, 0.05 significance) indicated that to be able to detect 20% increase in survival of Bcs1l mutant mice in in-house C57BL/6BomTac-derived background we would need 5 mice per group, both genders included. In the end, we observed 300% increase in the survival with group sizes being 6 and 8. The sample sizes in biochemical analyses (enzyme activity assays, respirometry and Blue Native PAGE) were partly limited by the nature of the method. A post-hoc power analysis showed 82% and 92% power to be able detected the observed change in complex III activity in liver and kidney, respectively, in the Bcs1l mutant mice.

2. Data exclusions

Describe any data exclusions.

One kidney sample was excluded from respirometry data analysis. Respiratory control ratio of this sample was 4.6 SD away from group mean value and a recorded protocol deviation was found. One Blue Native PAGE kidney sample (30 samples analyzed) was excluded, before any data analyses, due to total protein staining indicating a technical artefact.

3. Replication

Describe the measures taken to verify the reproducibility of the experimental findings.

We analyzed two (respirometry, CI, CII, CIV activity) or four (CIII activity) tissues and repeated the respirometry, liver and kidney CIII activity assays and qPCR from an independent mouse panel to corroborate the robustness of the findings. We utilized maximal feasible sample sizes for most assays. Moreover, our in vivo data was backed up by molecular dynamics simulations and in vitro assay with purified enzymes. We provided detailed description of the methods to render independent replication possible. We are also willing to exchange protocols upon enquiry.

4. Randomization

Describe how samples/organisms/participants were allocated into experimental groups.

The Bcs1l genotypes were randomized by the Mendelian rules. The nature of mtDNA inheritance did not allow randomization of mt-Cyb genotypes. The experiment was designed so that other genetic factors were essentially fully controlled. The order of sample collection and fresh sample analyzes was dictate by the birth date of the mice. All stored samples were analyzed in computationally randomized order.

5. Blinding

Describe whether the investigators were blinded to group allocation during data collection and/or analysis.

The personnel of the animal facility evaluating the mice were unaware of the mt-Cyb genotype information. Due to striking size difference, Bcs1l mutant mice could be easily distinguished from wild-type littermates. The data were collected and analyzed without awareness to group allocation, though, no strict blinding was applied.

Note: all in vivo studies must report how sample size was determined and whether blinding and randomization were used.

November 2017

6. Statistical parameters

For all figures and tables that use statistical methods, confirm that the following items are present in relevant figure legends (or in the Methods section if additional space is needed).

n/a	Confirmed	
☐	☒	The exact sample size (*n*) for each experimental group/condition, given as a discrete number and unit of measurement (animals, litters, cultures, etc.)
☐	☒	A description of how samples were collected, noting whether measurements were taken from distinct samples or whether the same sample was measured repeatedly
☐	☒	A statement indicating how many times each experiment was replicated
☐	☒	The statistical test(s) used and whether they are one- or two-sided<br>*Only common tests should be described solely by name; describe more complex techniques in the Methods section.*
☐	☒	A description of any assumptions or corrections, such as an adjustment for multiple comparisons
☐	☒	Test values indicating whether an effect is present<br>*Provide confidence intervals or give results of significance tests (e.g. P values) as exact values whenever appropriate and with effect sizes noted.*
☐	☒	A clear description of statistics including central tendency (e.g. median, mean) and variation (e.g. standard deviation, interquartile range)
☐	☒	Clearly defined error bars in all relevant figure captions (with explicit mention of central tendency and variation)

See the web collection on statistics for biologists for further resources and guidance.

▸ Software

Policy information about availability of computer code

7. Software

Describe the software used to analyze the data in this study.	Statistical analyzed for in vivo data were performed using GraphPad Prism 7 (survival data) or IBM SPSS Statistics 24 (other analyses).

For manuscripts utilizing custom algorithms or software that are central to the paper but not yet described in the published literature, software must be made available to editors and reviewers upon request. We strongly encourage code deposition in a community repository (e.g. GitHub). *Nature Methods* guidance for providing algorithms and software for publication provides further information on this topic.

▸ Materials and reagents

Policy information about availability of materials

**8. Materials availability**	
Indicate whether there are restrictions on availability of unique materials or if these materials are only available for distribution by a third party.	No unique materials were used.
**9. Antibodies**	
Describe the antibodies used and how they were validated for use in the system under study (i.e. assay and species).	We used well established monoclonal antibodies validated for Blue Native PAGE. All antibodies were from MitoSciences /Abcam ltd.: RISP (clone, 5A5), CORE2 (13G12AF12BB11), NDUFA9 (20C11B11B11), ATP5A (15H4C4) and SDHB (21A11AE7).
**10. Eukaryotic cell lines**	
a. State the source of each eukaryotic cell line used.	No eukaryotic cell lines were used.
b. Describe the method of cell line authentication used.	No eukaryotic cell lines were used.
c. Report whether the cell lines were tested for mycoplasma contamination.	No eukaryotic cell lines were used.
d. If any of the cell lines used are listed in the database of commonly misidentified cell lines maintained by ICLAC, provide a scientific rationale for their use.	No commonly misidentified cell lines were used.

▸ Animals and human research participants

Policy information about studies involving animals; when reporting animal research, follow the ARRIVE guidelines

11. Description of research animals

Provide all relevant details on animals and/or animal-derived materials used in the study.	We used mice of commercial C57BL/6JCrl genetic background, a C57BL/6JBomTac-derived mouse strain and F1 hybrid mice from these two substrains. Balanced groups of both genders or if not possible, only males were used.

Policy information about studies involving human research participants

12. Description of human research participants

Describe the covariate-relevant population characteristics of the human research participants.	The study did not involve human participants.
